# A timely death of the tapetum underlies MORE FLORET1 (MOF1) regulation of male fertility in rice

**DOI:** 10.1093/plphys/kiae208

**Published:** 2024-04-09

**Authors:** Janlo M Robil

**Affiliations:** Assistant Features Editor, Plant Physiology, American Society of Plant Biologists; Department of Biology, School of Science and Engineering, Ateneo de Manila University, Quezon City 1108, Philippines

Hybrid cereal crops, developed by crossing parents with different genetic backgrounds, contribute to global food security through their superior vigor and yields. However, hybrid breeding is difficult to accomplish in self-pollinating crops, such as barley, wheat, and rice since it requires the use of male sterile lines lacking functional pollen to serve as female parents. For decades, breeding programs have been targeting this reproductive constraint that not only affects hybrid production in major crops but also impedes polyploidization strategies aimed at their improvement (Longin et al. 2012; Yu et al. 2021). Recently, Lu et al. (2020) discovered that mutation of the MYB transcription factor MORE FLORET1 (MOF1; Li et al. 2020; Ren et al. 2020) causes male sterility in tetraploid rice. This finding is significant because it uncovers a promising target for a novel hybridization system, which could potentially facilitate the development of polyploid hybrid rice.

MOF1 is essential for male fertility in rice, but precisely how the gene controls pollen production remains poorly understood. In this issue of *Plant Physiology*, Lu et al. (2024) addressed that question in both diploid and tetraploid rice. By analyzing *mof1* mutants and ectopic expression lines, the authors defined the tissue- and stage-specific activities of MOF1 during anther development. They found that MOF1 plays a role in the timely degeneration of a key anther tissue that determines pollen viability. In addition, they found that MOF1 acts as a repressor and controls the expression of a gene involved in pollen wall formation. Therefore, Lu et al. (2024) elucidate the developmental and molecular bases of MOF1 function, which are crucial for its potential utility as a male sterility system.

Expanding on their previous work in tetraploid rice (Lu et al. 2020), the authors investigated MOF1 function in diploid rice using CRISPR/Cas9-mediated *mof1* knockouts. Consistent with their hypothesis, diploid *mof1* mutants displayed significantly reduced pollen viability and seed-set. Interestingly, not only was male sterility conserved between diploid and tetraploid *mof1* mutants, but the defects in their spikelet morphology were also consistent, indicating that the suite of developmental roles of MOF1 is conserved across these ploidies (Fig. A). Lu et al. (2024) thus provide evidence that MOF1 plays a role in male fertility and, more broadly, in rice reproductive development across both diploid and tetraploid rice varieties.

**Figure.**
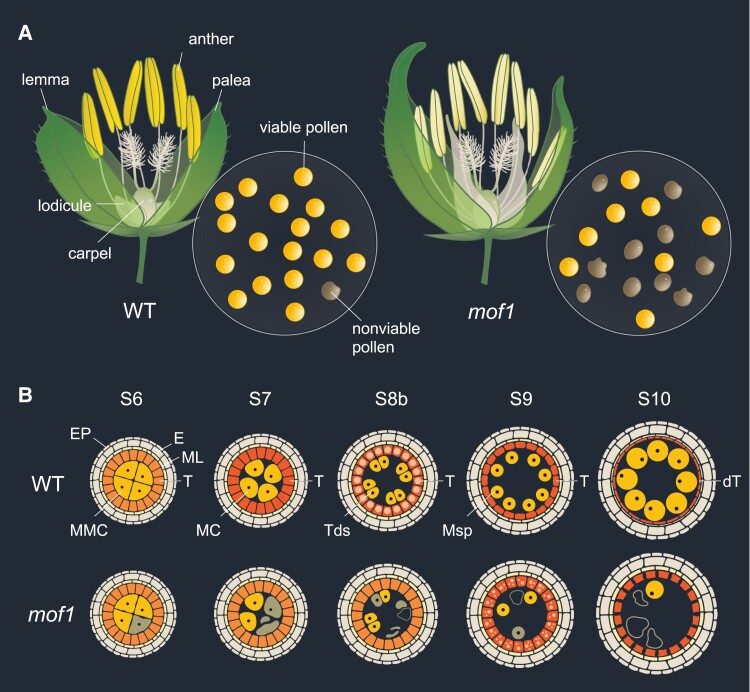
*MOF1* regulates spikelet development and male fertility in rice. **A)** Schematic diagram of spikelet morphology and pollen viability rate in wild type (WT) and *mof1* mutant. The *mof1* phenotypes include spikelets with abnormal lemma and palea, and florets with unusual size and number of floral structures. *mof1* mutants exhibit significantly reduced pollen fertility. **B)** Diagram of cross-sections of a developing rice pollen sac showing the progressive degeneration of the tapetum (T; orange) during microspore (Msp) formation (stages adapted from Zhang et al. 2011). Lu et al. (2024) found that *mof1* mutants exhibit a delay in tapetum degeneration, correlating with impaired pollen development and reduced viability. Abbreviations: dT, degenerated tapetum; E, endothecium; EP, epidermis; MC, meiotic cell; ML, middle layers; MMC, microspore mother cell; T, tapetum; Tds, tetrads.

The anther wall plays pivotal roles in pollen development, providing essential support and nourishment. The authors observed defects in anther wall development in both diploid and tetraploid *mof1* mutants, which correlated with the production of impaired nonviable pollen grains. While the outer layers of the anther wall formed normally, the middle layers and the tapetum exhibited delayed degeneration in *mof1* mutants (Fig. A). The tapetum is the innermost layer of the anther wall, which degenerates via programmed cell death to excrete nutrients and structural substances. Retardation of this process often leads to impaired pollen development, as seen in various male sterility mutants in maize, rice, and Arabidopsis (Marchant and Walbot 2022). Therefore, Lu et al. (2024) concluded that MOF1 regulates tapetum degeneration in rice anthers, which could underlie the male sterility phenotype in *mof1* mutants.

Is the timing of *MOF1* expression important for tapetum degeneration and pollen development? To address that question, Lu et al. (2024) created transgenic lines that ectopically express *MOF1* in the tapetum at a later stage of anther development using the ETERNAL TAPETUM1 promoter (Niu et al. 2013). In these transgenic lines, MOF1 expression peaked later than normal. Although the spikelets developed normally, the anthers exhibited delayed tapetum degeneration and harbored a significant number of aborted pollen grains. These findings suggest that the timely decrease of MOF1 expression during a specific stage drives tapetum degeneration, which is crucial for the production of viable pollen grains.

To dissect the regulatory activity of MOF1 in the tapetum, the authors examined 5 tapetal genes that are downregulated in tetraploid *mof1* mutants (Lu et al. 2020). They knocked out these individual genes using CRISPR/Cas9, and the resulting mutant lines exhibited delayed tapetum degeneration, abnormal pollen development, and sterility. When the expression levels of these 5 genes and *MOF1* were compared throughout anther development, a striking reciprocity was observed. *MOF1* expression peaked earlier in development, followed by a significant decline. In contrast, the 5 genes displayed an opposite pattern, with their expression levels increasing later and peaking at a different time point. They later confirmed that MOF1 directly represses the expression of 1 of these tapetal genes, which is involved in lipid and phenolic metabolism. Therefore, Lu et al. (2024) concluded that MOF1 negatively regulates tapetal genes to prevent their premature expression during anther development and facilitate normal pollen development.

Beyond its application in plant breeding, the work of Lu et al. (2024) opens exciting new avenues in plant reproductive biology. This work and previous studies (Li et al. 2020; Lu et al. 2020; Ren et al. 2020) revealed pleiotropic floral defects and altered gene expression profiles in both diploid and tetraploid *mof1* mutants. These findings suggest that MOF1 could be a key player in various pathways regulating flower development and function in rice. As such, future research can explore the function and evolution of MOF1 between wild and domesticated species and across major cereal clades. Moreover, single-cell multi-omics approaches could help map out MOP1 regulatory networks, from floral meristem development to pollen production. By targeting specific modules or pathways, it would then be possible to engineer precision hybridization systems, enabling the production of new high-yielding and stress-resistant hybrid varieties of cereal crops.
